# Supplementary material for: Coseismic fault sealing and fluid pressurization during earthquakes
Source: Nat Commun. 2023 Mar 8;14:1136. doi: 10.1038/s41467-023-36839-9 (PMC9995344; doi:10.1038/s41467-023-36839-9)
Supplement: Supplementary file 1 — Supplementary Information [file 41467_2023_36839_MOESM1_ESM.pdf]

Supplementary Information for "Coseismic fault sealing and fluid pressurization during earthquakes"

Lu Yao[1]*, Shengli Ma[1], Giulio Di Toro[2,3]

1. State Key Laboratory of Earthquake Dynamics, Institute of Geology, China Earthquake Administration, Beijing, China.
2. Dipartimento di Geoscienze, University of Padua, Padua, Italy.
3. HPHT Laboratory, INGV, Rome, Italy.

*Corresponding author: Lu Yao (luyao@ies.ac.cn)

This file contains additional information about experimental configuration, experimental results, postmortem microstructures, and numerical modeling of thermal pressurization that considers wear-induced sealing effects.

Contents of the file:

1. Supplementary Figures S1 to S7

2. Supplementary Tables S1 to S2

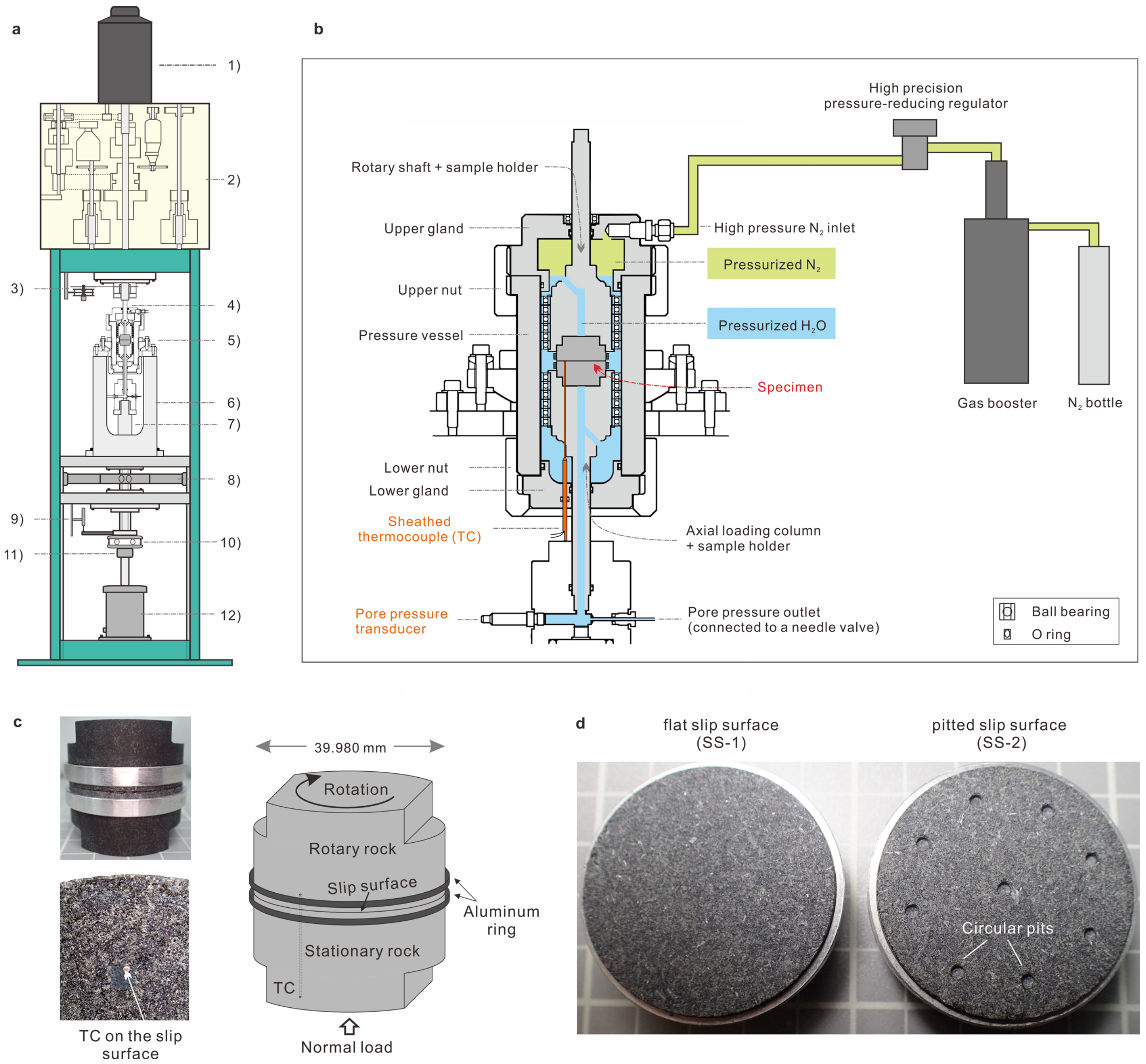

Supplementary Figure S1: Experimental setup and rock specimens. a Sketch of the low- to high-velocity frictional testing machine equipped with a room-temperature pressure vessel at Institute of Geology, China Earthquake Administration[1,2]. 1) servomotor, 2) gear and belt system for changing slip velocity, 3) rotary encoder and potentiometer, 4) rotary shaft, 5) pressure vessel, 6) metal frame for fixing the pressure vessel, 7) axial loading column, 8) cantilever-type torque gauge, 9) axial displacement transducer, 10) thrust bearing, 11) axial force gauge, and 12) air actuator. **b** Magnified view of the pressure vessel with main parts indicated in the diagram[2]. For a given set point, the poppet valve inside the pressure-reducing regulator would be closed if the downstream pressure increases, making a closed system of the vessel in the experiments. **c** Photo and schematic diagram of a pair of dolerite samples for the experiments. The tip of the sheathed thermocouple is located 15 mm from the center of the slip surface. **d** Photo showing flat and pitted slip surfaces prepared for the experiments. Only the stationary block has pits (one pit in the center and the other 8 pits located 14 mm from the center) in the experiments with the pitted slip surface.

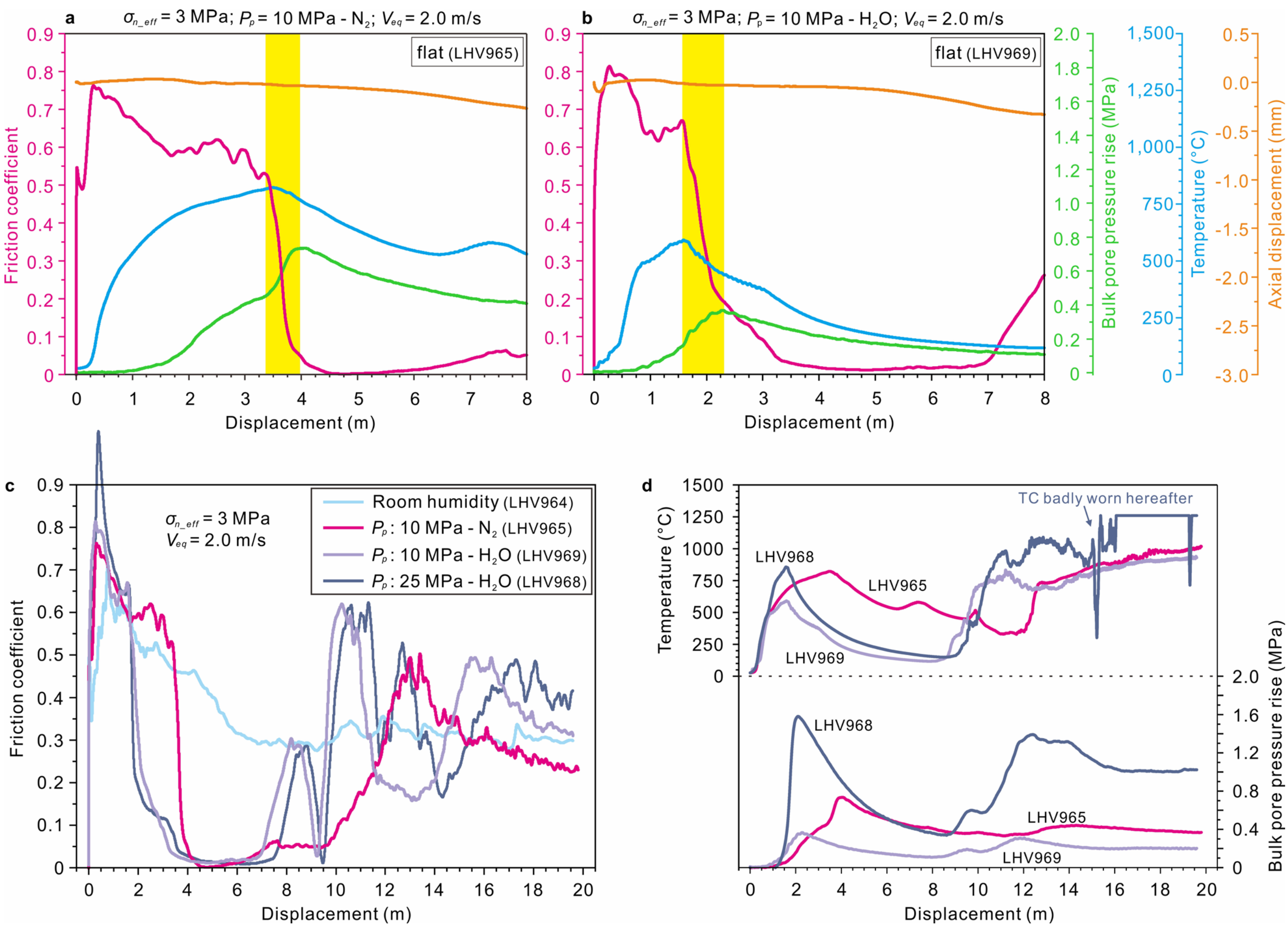

Supplementary Figure S2: Results from the experiments performed under effective normal stress (σ_{n_eff}) of 3 MPa, slip rate (V_{eq}) of 2.0 m/s, and pore pressure (P_p) of 10 and 25 MPa. a and **b**, The evolution of friction, pore pressure rise, temperature and axial displacement during the first 8 m slip. The yellow shading highlights the transient sharp weakening (TSW) occurring concurrently with the spike in pore fluid pressure. **c** and **d,** The whole picture of the evolution of friction, temperature and bulk pore pressure rise during 20 m slip. The TSW, frictionless sliding and re-strengthening only occur in the presence of pressurized pore fluids (cp. the room humidity case and the pore fluid cases in **c**). The minor differences in thermal expansivity, compressibility and viscosity between H_2O and N_2 at temperatures higher than ~500 °C may explain the quite similar TSW in the cases of these two pore fluids. The large fluctuation in friction during the re-strengthening stage in the cases of pore water pressure (LHV968 and LHV969) is probably caused by the complicated fracturing (probably promoted by quenching in the surrounding cool water), wear-induced sealing, and bulk melting processes in the presence of water. The temperature evolution was monitored using the sheathed thermocouples exposing on the slip surfaces. The thermocouple was probably badly worn after 15 m slip in the run LHV968 (**d**). The convergence of the temperature data after ~15 m slip may suggest the occurrence of bulk melting.

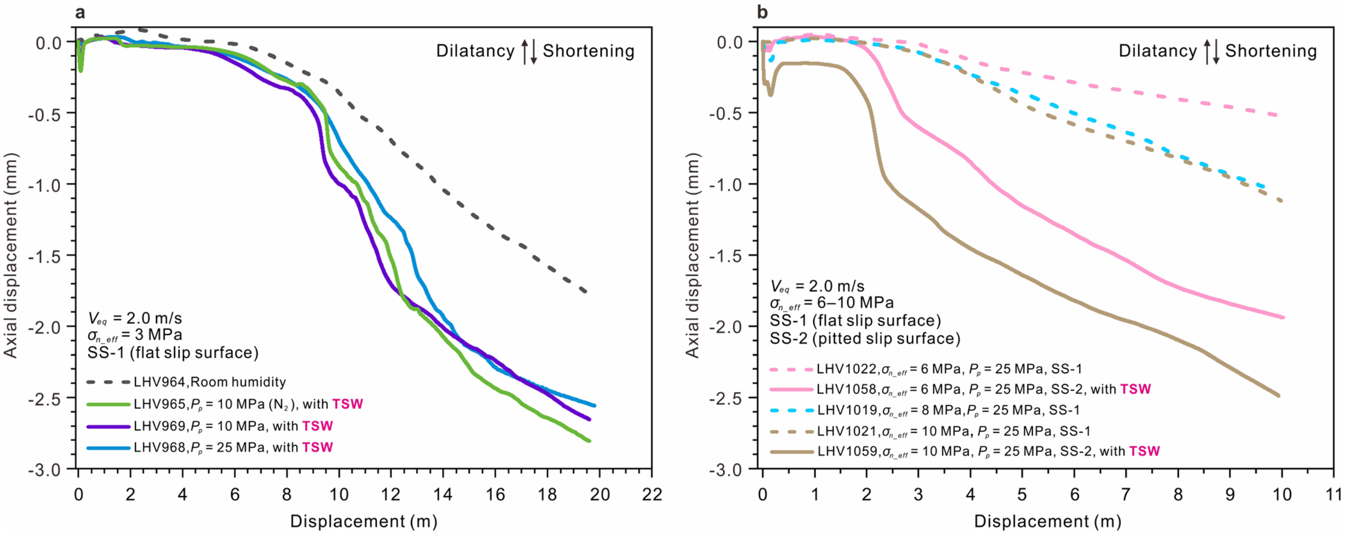

Supplementary Figure S3: Compiled data of evolution of axial displacement with slip displacement. **a** Experiments performed under effective normal stress (σ_{n_eff}) of 3 MPa, slip rate (V_{eq}) of 2.0 m/s, and pore pressure (P_p) of 10 or 25 MPa (flat slip surfaces, SS-1, were used). **b** Experiments performed under σ_{n_eff} = 6–10 MPa, V_{eq} = 2.0 m/s, and P_p = 25 MPa (both flat and pitted (SS-2) slip surfaces were used). The experiments showing transient sharp weakening (TSW; solid lines) tend to have significant increases in shortening rate after the TSW stages.

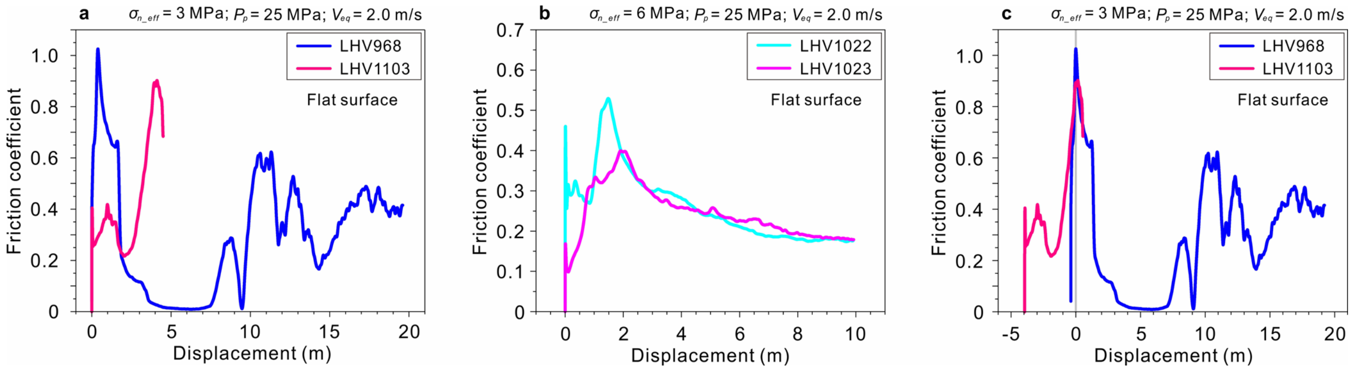

Supplementary Figure S4: Comparison of mechanical data from several experiments performed under effective normal stress (σ_{n_eff}) of 3 (a and c) or 6 (b) MPa, slip rate (V_{eq}) of 2.0 m/s, and pore pressure (P_p) of 25 MPa. The friction evolution prior to the peak friction is complicated in the experiments LHV1103, LHV1022 and LHV1023 (**a** and **b**; also in tests LHV1058, LHV1021 and LHV1059 as shown in Figs. 2c and 2d). The presence of multiple friction peaks and the scatter in the friction data during the initial acceleration and slip is quite common for solid cylindrical samples due to the misalignment of the two rock cylinders[3,4] and the complicated thermal evolution of the slip zones[5,6]. Once the initial evolution is overcome, and the friction decays towards the so-called "steady-state" conditions with good reproducibility [4] (see also in **b**), especially in the absence of pressurized fluids. By shifting the displacement at which the friction reaches the peak to 0 m for the tests LHV968 and LHV1103 (**c**), the data suggest that the run LHV1103 was terminated at the onset or initial stage of the transient sharp weakening (TSW).

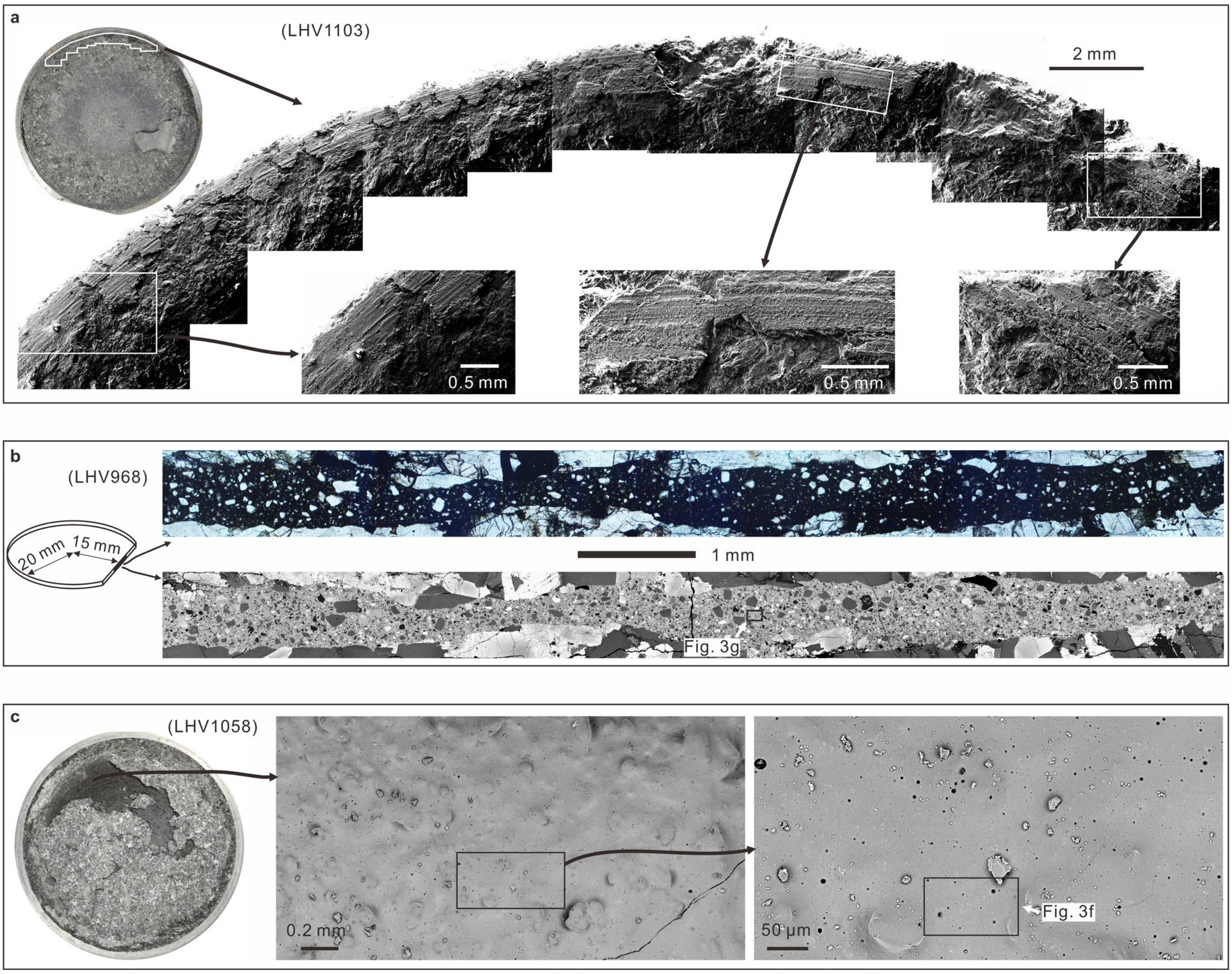

Supplementary Figure S5: Deformation textures of the samples recovered from three experiments. **a** Ring-shaped slickensides developed on the slip surface of the test LHV1103 (secondary electron images). **b** Plane-polarized optical micrograph and back-scattered electron image of the continuous molten layer formed in the run LHV968 (the location of the thin-section is indicated in the figure). **c**, Photograph and secondary electron images of the slip surface after the test LHV1058. The experimental conditions under which the samples were deformed are listed in Supplementary Table S1.

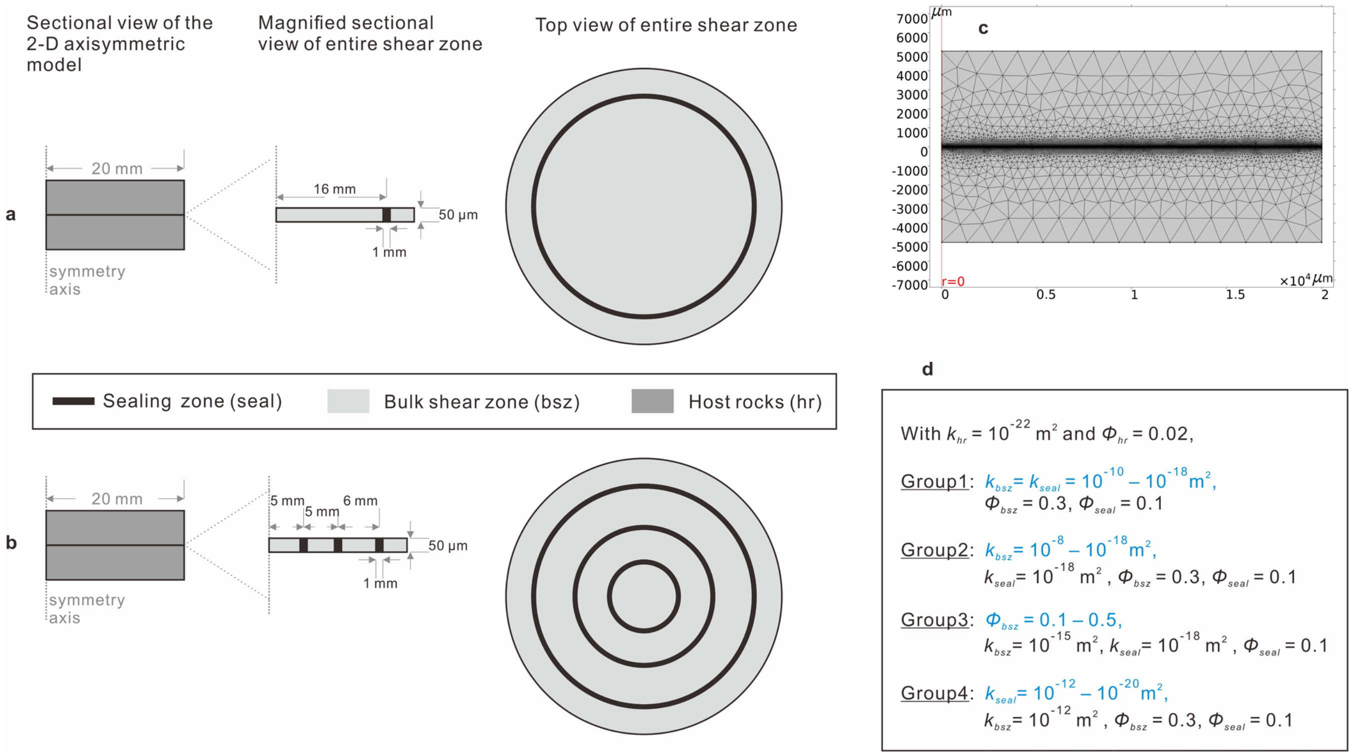

Supplementary Figure S6: **Details of thermal pressurization modeling**. **a** and **b**, Sectional and top view of the 2-D axisymmetric finite element models for thermal pressurization modeling with Comsol Multiphysics. Two kinds of geometric models, with either one (**a**) or three (**b**) ring-shaped sealing zones, were evaluated here. **c** Mesh model for the modeling. **d** Four groups of parameter settings of permeability (*k*) and porosity (φ) are used to examine how the existence of sealing zone(s) may aid TP weakening and how higher amount of water may facilitate transient TP in the experiments. The subscripts ‘bsz’, ‘seal’ and ‘hr’ are abbreviations of bulk shear zone, sealing zone and host rock, respectively.

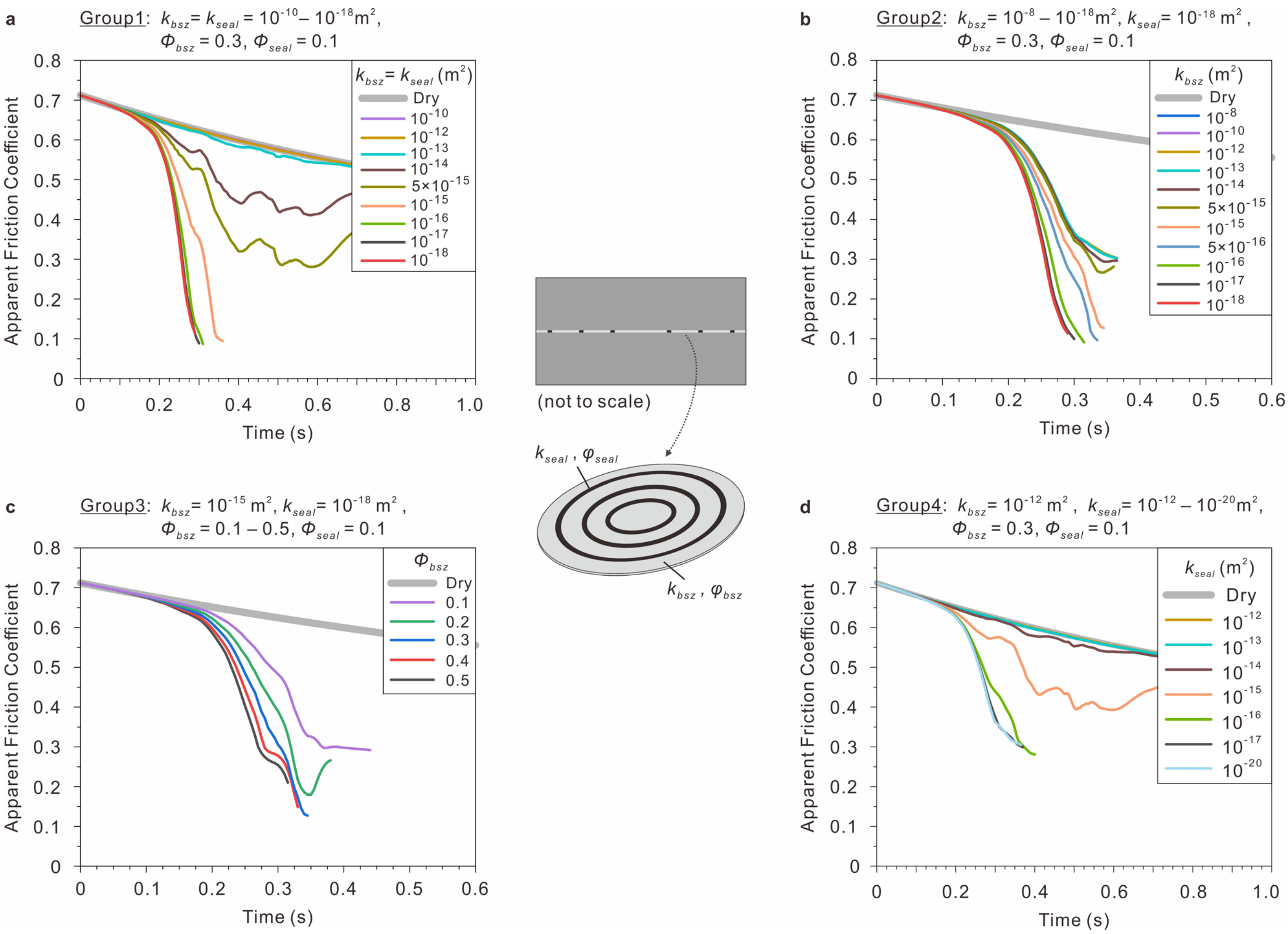

Supplementary Figure S7: Results from thermal pressurization modeling with the geometric model containing three ring-shaped sealing zones. **a–d** Counterparts of the results shown in Figs. 4a–d, respectively (see Fig. S6b).

Supplementary Table S1: Summary of friction experiments.

Run No.	Effective normal stress, σ_{n_eff} (MPa)	Environmental conditions / Pore fluid	Pore pressure, P_p (MPa)	Equivalent slip rate, V (m/s)	Total displacement, d (m)	Slip surface
LHV964	3	Room humidity	/	2	20	flat
LHV965	3	High purity N_2	10	2	20	flat
LHV969	3	Deionized H_2O	10	2	20	flat
LHV968	3	Deionized H_2O	25	2	20	flat
LHV1103	3	Deionized H_2O	25	2	4.5	flat
LHV1022	6	Deionized H_2O	25	2	10	flat
LHV1023	6	Deionized H_2O	25	2	10	flat
LHV1058	6	Deionized H_2O	25	2	10	pitted
LHV1021	10	Deionized H_2O	25	2	10	flat
LHV1059	10	Deionized H_2O	25	2	10	pitted

Supplementary Table S2: Thermal, hydraulic, and other physical properties used in thermal pressurization modeling. Most of the parameters follow those used in refs. 2 and 7 and references therein. The temperature- and pressure-dependent properties of water were downloaded from NIST Standard Reference Database.

**Parameter**	**Symbol**	**Value/Expression**	**Unit**
Thickness of shear zone	W	50	μm
Radius of host blocks	r_0	20	mm
Effective normal stress	σ_{n_eff}	3	MPa
Initial pore pressure	P_p	25	MPa
Porosity	Φ		
Bulk shear zone	Φ_{bzs}	0.1 to 0.5	1
Sealing zone	Φ_{seal}	0.1	1
Dolerite	Φ_{hr}	0.02	1
Permeability	k		
Bulk shear zone	k_{bzs}	1×10^{-8} to 1×10^{-18}	m^2
Sealing zone	k_{seal}	1×10^{-10} to 1×10^{-20}	m^2
Dolerite	k_{hr}	1×10^{-22}	m^2
Density	ρ		
Mineral grains	ρ_m	2800	kg/m^3
Pore water	ρ_f	T- & P-dependent	kg/m^3
Shear zone, sealing zone & dolerite		$\rho_m (1-\Phi) + \rho_f\Phi$	kg/m^3
Thermal conductivity	λ		
Mineral grains	λ_m	3.5	W/m/k
Pore water	λ_f	T- & P-dependent	W/m/k
Shear zone, sealing zone & dolerite		$\lambda_m^{(1-\Phi)} \lambda_f^{\Phi}$	W/m/k
Specific heat capacity	C_p		
Mineral grains	C_{p_m}	750	J/kg/k
Pore water	C_{p_f}	T- & P-dependent	J/kg/k
Shear zone, sealing zone & dolerite		$[\rho_m C_{p\_m} (1-\Phi)+ \rho_f C_{p\_f}\Phi]/\rho$	J/kg/k
Thermal expansivity (volumetric)			
Pore water	α_f	T- & P-dependent	k^{-1}
Mineral grains	α_m	2.2×10^{-5}	k^{-1}
Compressibility			
Mineral grains	β_m	1.2×10^{-11}	Pa^{-1}
Pore water	β_f	T- & P-dependent	Pa^{-1}
Dolerite (bulk compressibility)	β_b	2×10^{-10}	Pa^{-1}
Shear zone (bulk compressibility)	β_b	2×10^{-8}	Pa^{-1}
Sealing zone (bulk compressibility)	β_b	2×10^{-8}	Pa^{-1}
Dynamic viscosity			
Pore water	η	T- & P-dependent	Pa·s

References

1. Ma, S., Shimamoto, T., Yao, L., Togo, T. & Kitajima, H. A rotary-shear low to high-velocity friction apparatus in Beijing to study rock friction at plate to seismic slip rates. *Earthquake Sci.* **27**, 469–497, (2014).
2. Yao, L., Ma, S., Chen, J., Shimamoto, T. & He, H. Flash heating and local fluid pressurization lead to rapid weakening in water-saturated fault gouges. *J. Geophys. Res.* **123**, 9084–9100, (2018).
3. Hirose, T. & Shimamoto, T. Growth of molten zone as a mechanism of slip weakening of simulated faults in gabbro during frictional melting. *J. Geophys. Res.* **110**, B05202, (2005).
4. Niemeijer, A., Di Toro, G., Nielsen, S. & Di Felice, F. Frictional melting of gabbro under extreme experimental conditions of normal stress, acceleration, and sliding velocity. *J. Geophys. Res.* **116**, B07404, (2011).
5. Fialko, Y. & Khazan, Y. Fusion by earthquake fault friction: Stick or slip? *J. Geophys. Res.* **110**, B12407 (2005).
6. Nielsen, S., Di Toro, G., Hirose, T. & Shimamoto, T. Frictional melt and seismic slip. *J. Geophys. Res.* **113**, B01308, (2008).
7. Oohashi, K., Hirose, T., Takahashi, M. & Tanikawa, W. Dynamic weakening of smectite-bearing faults at intermediate velocities: Implications for subduction zone earthquakes. *J. Geophys. Res.* **120**, 1572–1586, (2015).
